# Review on Eco-Environment Research in the Yellow River Basin: A Bibliometric Perspective

**DOI:** 10.3390/ijerph191911986

**Published:** 2022-09-22

**Authors:** Lina Liu, Jingjing Zeng, Xinnian Wu, Jiansheng Qu, Xuemei Li, Jing Zhang, Jinyu Han

**Affiliations:** 1 Northwest Institute of Eco-Environment and Resources, Chinese Academy of Sciences, Lanzhou 730000, China; zengjj@llas.ac.cn (J.Z.); wuxn@lzb.ac.cn (X.W.); zhangj@llas.ac.cn (J.Z.); 2 Chengdu Library and Information Center, Chinese Academy of Sciences, Chengdu 610041, China; jsqu@lzb.ac.cn (J.Q.); hanjinyu00000@163.com (J.H.); 3 China University of Political Science and Law Library, Beijing 100088, China; lixuemei0211@cupl.edu.cn

**Keywords:** Yellow River Basin, ecological environment, bibliometric, research review

## Abstract

The Yellow River Basin (YRB) is an important economic zone and ecological barrier in China. The analysis of its research characteristics and hotspots has been helpful to grasping the future research direction. This work carried out text mining and analysis on scientific papers related to eco-environment research in the YRB from English and Chinese publications. It showed that: there was a fluctuating upward trend over the past 30 years, which was closely related to major events in the YRB during the same period. Chinese research institutions have a closer cooperation with the USA, Australia and other developed countries. More articles were from high-quality journals in ecology, the environment, and others. Interestingly, research institutions with more Chinese articles were mainly located around Beijing or the YRB. Additionally, from a research object perspective, both the English and Chinese articles have mainly focused on large areas such as the lower Yellow River, the middle reaches of the Yellow River, and the upper reaches of the Yellow River, then turning to small areas such as the Yellow River estuary and the source area of the Yellow River. Eco-environment research in the YRB has involved multiple disciplines, and “water–soil–vegetation–ecological protection” has been widely concerned. From the evolution law of hot topics, it has shown a transformation from quantity to quality, from utilization to management, from macro to micro, from construction to high-quality development. It suggests that future research should focus on water, soil, the ecological environment and local high-quality development in small regions and small watersheds.

## 1. Introduction

The Yellow River Basin (YRB) is a vast territory, spanning four geomorphic units from west to east across the Qinghai–Tibet Plateau, Inner Mongolia Plateau, Loess Plateau and Yellow Huaihai Plain [1,2]. It contained a large population as of the end of 2019, with a total permanent population of about 422 million and a GDP of 24.74 trillion RMB, accounting for about 36.91% and 25.11% of the total, respectively [3]. The proposal of “Outline Document on Ecological Protection and High-Quality Development of the Yellow River Basin” to firmly follow the path of green, sustainable and high-quality development has received extensive attention from local governments and researchers [3]. The YRB is an important economic zone and ecological barrier in China that bears the ecological stress caused by rapid urbanization and extensive industrialization, and it is highly representative in its green development and ecological governance [4,5,6].

Strengthening the ecological protection and high-quality development of YRB is not only related to national ecological security, but also has an important strategic position for China’s future economic development [7,8,9]. However, the eco-environmental problems in YRB are very prominent, such as serious shortage of water resources, high utilization rate of development, and difficulty in guaranteeing the eco-environment [10,11,12]; some regions have poor environmental quality and have been difficult to improve [13,14,15,16]; there has been degradation of the ecosystem and a decline in service functions [17,18]; the potential risk of eco-environment is high and easy to transform into social risk [19,20,21,22].

Mining the text data of the relevant publications based on bibliometric data was helpful to clarify the situation and develop the trend of research, which also provided a reference for future research [23,24,25]. Lu et al. analyzed the research status of agricultural irrigation in the YRB based on bibliometric data and found that the ecological pollution problem, advanced irrigation and drainage technologies in the irrigation areas were the main research directions for the future [24]. Zhang et al. analyzed the frozen soil research in the YRB and found that the permafrost, climate change and ecological environment in the source area of the Yellow River (YR) were the main research topics [17]. Wohlfart et al. [23] and He et al. [25] showed that water scarcity and the eco-environment pollution and degradation has been aggravated by human activities and the related climate change using a bibliometric method. The existing research results provide scientific theoretical support and academic contributions of the eco-environment research in the YRB in the new research period.

The bibliometric analysis of eco-environment research in the YRB can be expanded into the following two aspects. Firstly, there were some uncertainties in the results of the bibliometric analysis due to its different sources of data collection and retrieval strategies [25,26,27]. For example, the YRB was only used as the theme to retrieve the key research areas and topics, and much irrelevant information appeared. It was necessary to consider the retrieval strategy and authoritative multi-data sources of scientific papers on eco-environment research in the YRB [17,26]. Additionally, in the analysis of the research hotspots, the existing studies often explored the thematic analysis from the initial year to the termination year, which inevitably ignored the comparative analysis of different periods. A comparative analysis of different time periods can better grasp the changing trends of eco-environment research topics and frontiers in the YRB [25,27]. Hence, this work used Web of Science (WoS) and China National Knowledge Infrastructure (CNKI) as the data sources, and used the software CiteSpace [28,29] and VOSviewer [30] as tools to conduct text mining and visual analysis, which enriched the data sources and suggested future directions for eco-environment research in the YRB.

Over the past thirty years, research on the eco-environment in the YRB has shown an overall upward trend, suggesting the growing awareness and recognition of it. However, systematic review papers on it are relatively scarce. Hence, this work mainly adopted a bibliometric analysis to summarize its research characteristics and hotspots and to discuss its evolution process. This study answered the following questions: (1) what is characteristic of the scientific literature on eco-environment research in the YRB; (2) and what are the future research trends. The results based on answering the above questions effectively grasped the hot frontiers and key issues of eco-environment research and provided new ideas for ecological protection and high-quality development in the YRB.

## 2. Data Collection and Methods

The WoS platform includes thousands of multidisciplinary international academic journals, and journals from the Science Citation Index Expanded (SCIE) and Social Science Citation Index (SSCI) are widely influential. For the Chinese literature retrieval, the journals included the Chinese Science Citation Database (CSCD), Chinese Social Sciences Citation Index (CSSCI), and Core Journals (Core) from CNKI, which have strict standards and a wide influence. The articles in English and Chinese were obtained from WoS and CNKI databases, respectively.

**Figure 1:**
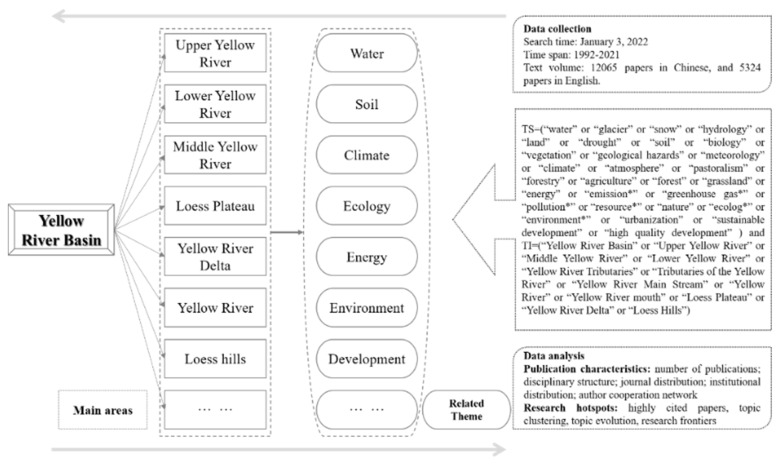
The framework of research analysis process.

In order to better grasp the research hotspots, this work adopted the following retrieval strategies (Figure 1): (1) first, extracting the key research areas and topics with the YRB as the theme; (2) searching articles with the main research regions and topics as the titles and themes; (3) identifying the search methods based on the research areas and topics through several attempts; (4) finally, determining the retrieval formula, which was “TS equal (“water” or “glacier” or “snow” or “hydrology” or “land” or “drought” or “soil” or “biology” or “vegetation” or “geological hazards” or “meteorology” or “climate” or “atmosphere” or “pastoralism” or “forestry” or “agriculture” or “forest” or “grassland” or “energy” or “emission*” or “greenhouse gas*” or “pollution*” or “resource*” or “nature” or “ecolog*” or “environment*” or “urbanization” or “sustainable development” or “high-quality development”) and TI equal (“Yellow River Basin” or “Upper Yellow River” or “Middle Yellow River” or “Lower Yellow River” or “Yellow River Tributaries” or “Tributaries of the Yellow River” or “Yellow River Main Stream” or “Yellow River” or “Yellow River mouth” or “Loess Plateau” or “Yellow River Delta” or “Loess Hills”); and LA = (English)); and DT = (Article)); and Citation Index = (SCI or SSCI in WoS; CSCD or CSSCI or Core in CNKI); and Time span = 1992–2021” within English and Chinese, respectively.

Based on the above retrieval method, it aimed to ensure that the retrieved scientific papers were more representative to a greater extent. The data were retrieved on 3 January 2022. Then, we cleaned the information, such as the countries and keywords by DDA, and removed some irrelevant information by reading the related abstract, keywords or results. Finally, 5324 English scientific papers and 12,065 Chinese scientific papers were used for the bibliometric analysis. This work explored the text mining and data analysis of eco-environment research in the YRB by using DDA software [31], CiteSpace 6.1.R2 (https://citespace.podia.com (accessed on 28 May 2022)) and VOSviewer version 1.6.18 (Centre for Science and Technology Studies, Leiden University, Leiden, the Netherlands).

## 3. Results

### 3.1. Characteristics of Articles

#### 3.1.1. Time-Series Characteristics

For the time-series characteristics from 1992 to 2021, the overall number of English articles on eco-environment research in the YRB showed a gradual upward trend. The number of Chinese articles showed a rising trend of fluctuation (“rising–falling–rising”).

- First bullet; English articles

**Figure 2:**
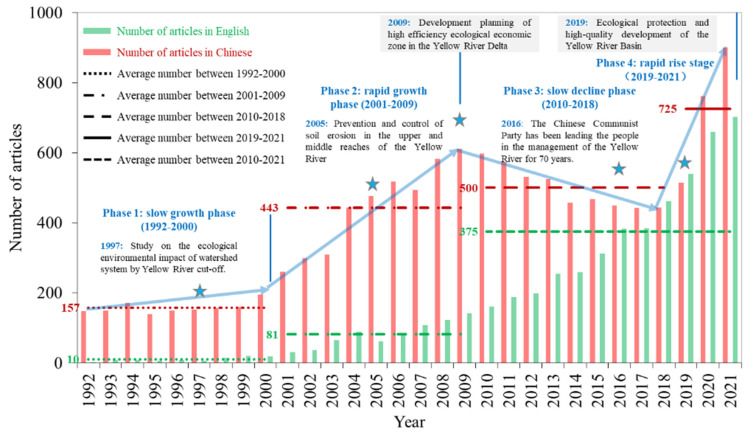
The number of annual research articles from 1992 to 2021.

It was found that the number of related English articles increased from 2 in 1992 to 703 in 2021, with a cumulative total of 5324 from WoS (Figure 2). The trend of articles could be divided into three stages. The first phase from 1992 to 2000 was considered an initial stage. The number of articles only accounted for 1.8% of the total, with an average of 10. By 2000, the number of cumulative articles was only 94. Phase II, 2001–2009, was considered a slow growth stage. The related articles accounted for 13.8% of the total, with an average of 81. The number of cumulative articles was 826 in 2009, 3.6 times higher than that in 2000. The third stage from 2010 to 2021 was considered to be a rapid growth period. The number of articles accounted for 84.5% of the total, with an average of 375. The number of cumulative articles was 4498 from 2010 to 2021, which was 5.5 times higher than that from 1992 to 2009. During the last 30 years, the overall characteristics of eco-environment research in the YRB has been increasing, revealing that the research on this topic has received widespread attention.

- Second bullet; Chinese articles

It was found that the number of related Chinese articles increased from 147 in 1992 to 900 in 2021, with a cumulative total of 12,065 from CNKI (Figure 2). The trend of articles could be divided into four stages, with a “rising–falling–rising” trend. Interestingly, the research trend was closely related to the major events and policies of ecological governance in the YRB [32]. In the first phase, the slow upward stage (1992–2000), the number of cumulative articles was 1416, with an average of 157, accounting for 11.7% of the total. During this period, the Chinese government began to study the impact of the Yellow River break on the eco-environment of the watershed system. The second phase was the rapid growth stage (2001–2009). The number of cumulative articles was 3988, with an average of 443, accounting for 33.0% of the total. During this period, the Chinese government paid more attention to the prevention and control of soil erosion in the middle and upper reaches of the Yellow River. In 2009, the National Development and Reform Commission of China issued a notice “the Development Plan of Yellow River Delta Efficient Ecological Economic Zone”. In this context, the number of the related articles reached a small peak. In the third phase, the slow fluctuation decline phase (2010–2018), the number of cumulative articles was 4485, with an average of 500, accounting for 41.4% of the total. The trend of this stage was closely related to the remarkable improvement of the YRB environment. In the fourth stage, the rapid growth stage (2019–2021), the number of cumulative articles was 2176, with an average of 725, accounting for 13.8% of the total. This showed that as the YRB’s ecological protection and high-quality development has risen to the level of national strategy, research on this subject has attracted wide attention in academia.

#### 3.1.2. The National Research Contribution

**Figure 3:**
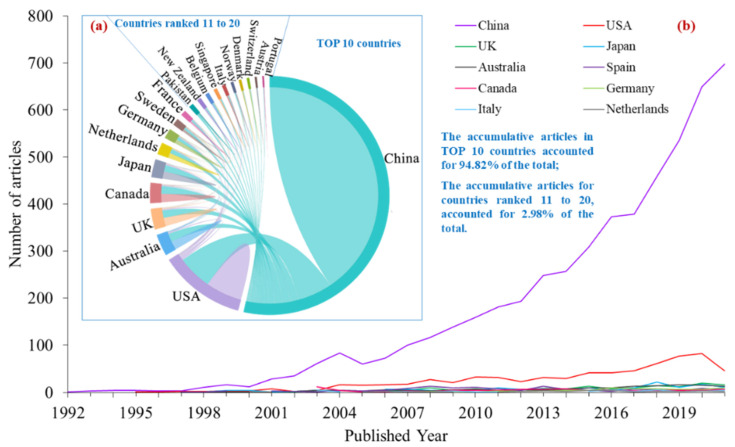
The national research contributions. (**a**) top 10 countries on this research; (**b**) Cooperation among top 20 countries/territories.

Since Chinese articles mainly covered one country, China, this section only analyzed the national research contribution from English articles. Over past 30 years, the related articles were from 62 different countries/territories. The number of cumulative articles in the top 10 countries accounted for 94.8% of the total; the top 11–20 countries accounted for 3.0% (Figure 3a). The YRB is located in China, which is one reason why Chinese scholars have studied this region the most. Other productive countries include the USA, Australia, the UK, Canada, Japan, the Netherlands, Germany, Sweden and France.

The national cooperation network diagram could effectively identify the intensity of cooperation among countries [28]. Based on DDA and the tubiaoxiu online drawing platform (www.tubiaoxiu.com (accessed on 28 May 2022)), this work drew the network diagram of the top 20 national/regional cooperation in terms of eco-environment research in the YRB (Figure 3b). It was found that China and the USA occupy a dominant position in terms of this research. In addition, Chinese scholars have cooperated closely with Australia, the UK, Canada, and Japan.

#### 3.1.3. Institutional Distribution Characteristics

- First bullet; English articles

There were 1760 research institutions that had published papers on eco-environment research in the YRB from WoS. A total of 1457 (82.8%) research institutions had published no more than 5 articles. Table 1 lists the top 10 most active research institutions. The Chinese Academy of Sciences (CAS) was in first place with 2161 articles, followed by Northwest A&F University with 1537, Ministry Water Resources with 563, Beijing Normal University with 482, Lanzhou University with 359, Xian University of Technology with 155, Ocean University of China with 153, Shaanxi Normal University with 131, Beijing Forestry University with 128, and China Institute of Water Resources and Hydropower Research with 121. The top 10 most active institutions accounted for 40.01% of the total number of articles. Meanwhile, it was found that the top 10 institutions were all from China, which also proved that China was active in eco-environment research in the YRB.

- Second bullet; Chinese articles

There were 7833 research institutions that had published papers on eco-environment research in the YRB from CNKI. A total of 6988 (89.2%) research institutions had published no more than 5 articles. As shown in Table 1, Northwest A&F University was in first place with 1643 articles, followed by Ministry Water Resources with 1088, Institute of Geographic Sciences Natural Resources Research CAS with 497, Yellow River Institute of Hydraulic Research, Yellow River Conservancy Commission with 448, Beijing Normal University with 413, University of Chinese Academy of Science CAS with 404, Lanzhou University with 397, Shaanxi Normal University with 309, Beijing Forestry University with 254, and Ocean University of China with 231. From the distribution of the top 10 research institutions, the overall concentration was relatively high, with 4 in Beijing, 3 in Shaanxi, and 1 in Gansu, Henan, and Shandong, respectively. Overall, the related research institutions were mainly located in Beijing or around the YRB.

#### 3.1.4. Discipline Structure Characteristics

- First bullet; English articles

**Figure 4:**
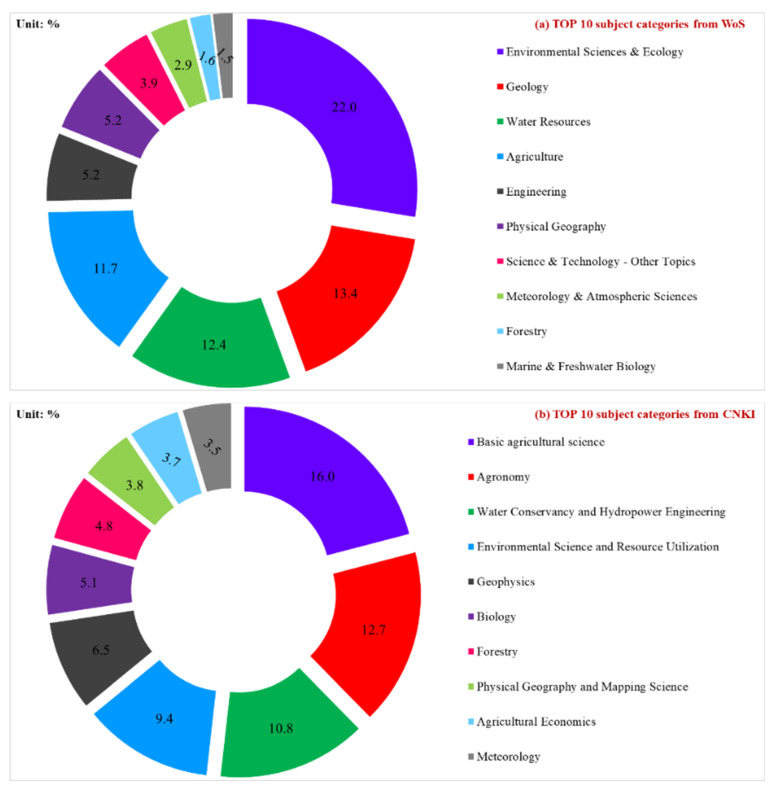
Top 10 disciplines for eco-environment research in YRB (**a**) from WoS and (**b**) from CNKI.

Based on the WoS database, eco-environment research in the YRB came from 72 disciplines. The top 10 disciplines were mainly distributed in environmental sciences and ecology (22.0%), geology (13.4%), water resources (12.4%), agriculture (11.7%), engineering (5.2%), physical geography (5.2%), science and technology—other topics (3.9%), meteorology and atmospheric sciences (2.9%), forestry (1.6%), marine and freshwater biology (1.5%), while others together accounted for the remaining 21.3% (Figure 4a). Eco-environment research in the YRB was an interdisciplinary research subject involving environmental science, ecology, geology, water resources science, climate change, and others. Therefore, eco-environment research in the YRB requires a comprehensive understanding of the overall knowledge of the environment, economy, society, and technology.

- Second bullet; Chinese articles

Based on the CNKI database, the top 10 disciplines were mainly distributed in basic agricultural science (16.0%), agronomy (12.7%), water conservancy and hydropower engineering (10.8%), environmental science and resource utilization (9.4%), geophysics (6.5%), biology (5.1%), forestry (4.8%), physical geography and mapping science (3.8%), agricultural economics (3.7%), meteorology (3.5%) and other disciplines (23.7%) (Figure 4b). It was found that the related Chinese articles also involved many disciplines. Overall, the research on eco-environment research in the YRB needs to be understood from the comprehensive and systematic perspective of “resources–ecology–environment–economy–society–technology”.

#### 3.1.5. Journal Distribution Characteristics

- First bullet; English articles

According to WoS, the related publications were published in 642 journals, but 454 journals published no more than 5 articles. Table 2 summarizes the top 10 most productive journals. Catena ranked first with 238 published articles (4.47%), followed by Science of the Total Environment (208, 3.91%) and Water (124, 2.33%). The top 10 most productive journals published 1194 articles, accounting for 22.4% of the total. It indicated that the concentration of journals published in related papers was high. The national sources of the top 10 journals were mainly the Netherlands, Switzerland, England, Germany and the USA. Among these top 10 most productive journals, four were published by Elsevier Publishing Group.

- Second bullet; Chinese articles

According to CNKI, the related publications were published in 833 journals. As shown in Table 2, the top 10 productive journals mainly included: Yellow River (1593, 13.2%), Bulletin of Soil and Water Conservation (448, 3.71%), Acta Ecologica Sinica (444, 3.21%), Research of Soil and Water Conservation (387, 3.21%), Journal of Soil and Water Conservation (359, 2.98%), Science of Soil and Water Conservation (233, 1.93%), Agricultural Research in the Arid Areas (229, 1.90%), Chinese Journal of Applied Ecology (208, 1.72%), Journal of Arid Land Resources and Environment (193, 1.60%), and Journal of Natural Resources (191, 1.58%). The results showed that the top 10 productive journals were mainly specialized journals related to eco-environment research in the YRB or professional journals focusing on resources, ecology and the environment. For example, the Journal of Natural Resources and Acta Ecologica Sinica are high-quality academic journals in the field of ecological and environmental research. From the perspective of the provinces where the top 10 journals are located, the overall concentration of journals was relatively high, with 4 in Shaanxi, 3 in Beijing, and 1 in Henan, Inner Mongolia, and Liaoning, respectively.

### 3.2. Research Hotspot Analysis

#### 3.2.1. Analysis of Highly Cited Papers

- First bullet; English articles

Generally, highly cited papers directly or indirectly reflect the research’s quality and influence, both globally and locally [26,27]. In this work, there were 41 highly cited papers from WoS on eco-environment research in the YRB (Supplementary Table S1). The analysis of these 41 highly cited papers showed that: the first rank received 673 citations and was published by Feng et al. (2016) in Nature Climate Change; this work focused on the vegetation reconstruction in the semi-arid Loess Plateau of China [33]. The second rank received 565 citations and was published by Wang et al. (2016) in Nature Geoscience; it was pointed that the erosion rates of the Loess Plateau would increasingly control the Yellow River’s sediment load [2]. The highly cited papers mainly focused on the Loess Plateau and Yellow River, and the research topics included human activities, groundwater quality, health risk assessment, climate change, soil moisture, soil erosion, etc. It was interesting that there were no highly cited papers before 2012, which was related to the fact that eco-environment research in the YRB was a regional issue and had not attracted much international attention. Relevant papers have been published in authoritative journals such as Nature Climate Change, Nature Communication, Nature Geoscience, Science of the Total Environment, Journal of Hydrology, and Catena.

- Second bullet; Chinese articles

According to the citation frequency ranking of CNKI, the retrieval of the top 20 highly cited papers on eco-environment research in the YRB is shown in Supplementary Table S2. The analysis of these top 20 highly cited papers showed that: three papers published by Bojie Fu et al. [34,35,36] on the interaction among land-use change, ecological environment, soil erosion and soil nutrients in the loess hilly sub-basin were studied. There were nine papers on loess hilly areas, eight of which were published between 1996 and 2004. This indicates that a series of problems such as soil, ecology and environment in the loess hilly region had been paid attention to earlier in China. Only one of the top 20 cited papers was published after 2013, namely, soil ecological stoichiometry under different vegetation areas in the loess hilly–gully region, which further indicates that soil and ecological problems in the loess hills and the surrounding areas had attracted much attention. The related papers have been published in authoritative journals such as Acta Geographica Sinica, Acta Ecologica Sinica, Journal of Natural Resources, Chinese Journal of Applied Ecology, Geographical Research, Chinese Science Bulletin and others.

#### 3.2.2. Keyword Cluster Analysis

- First bullet; English articles

**Figure 5:**
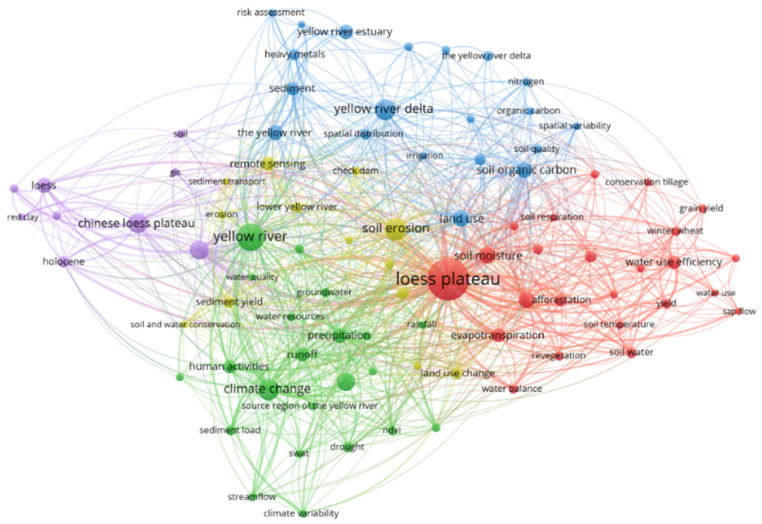
Keyword co-occurrence network map in the eco-environment research in YRB from WoS.

A bibliometric analysis of keywords could represent the research trends and frontiers of academic articles [28]. This work first provided a comprehensive picture of the related articles from WoS by keyword cluster analysis (Figure 5). The keyword network map of co-occurrence frequencies ≥20 from WoS was drawn. It was found that the research hotspots mainly focused on the four core regions of the Loess Plateau, the Yellow River, the Yellow River Delta and Chinese Loess Plateau. This work summarized the main topics of the related research in the past 30 years. It was found that: the main topics on the Loess Plateau were soil erosion, soil moisture, evapotranspiration, afforestation, water-use efficiency, and soil temperature. The research subject of the Yellow River mainly focused on climate change, human activities, runoff, precipitation, sediment load, drought, groundwater, and water quality. The research topics of the Yellow River Delta and Yellow River estuary were mainly focused on sediment, spatial distribution, irrigation, soil organic carbon, land use, and soil quality. For the Chinese Loess Plateau research areas, the migration of loess, red clay, Holocene, soil and sediment was mainly studied.

- Second bullet; Chinese articles

The keyword network map of co-occurrence frequencies ≥20 from CNKI was also drawn (Supplementary Figure S1). It was found that the research hotspots mainly focused on the four core regions of the Loess Plateau, the Yellow River Basin, the Yellow River Delta and the Yellow River. Summarizing the key themes, the results showed that: the research areas were mainly concentrated in the Loess Plateau and the Loess hilly and surrounding areas, and the research topics focused on soil moisture, soil nutrients, soil water content, vegetation restoration and biodiversity, species diversity, biomass and soil organic carbon. The research areas were mainly concentrated in the Yellow River, the middle reaches of the Lower Yellow River, the source region of the Yellow River, Xiaolangdi reservoir and the surrounding areas. In addition, the related research topics focused on the relationship between water resources, climate change, and human activities. The research areas were mainly concentrated in the Yellow River Delta and the Yellow River estuary. The related research topics were concerned with vegetation, soil, wetlands, heavy metals, environmental factors and other related issues. The research topics of the YRB were mainly concerned with ecological protection, ecological environment, soil conservation, sustainable development, high-quality development and other related issues.

#### 3.2.3. Theme Evolution Analysis

- First bullet; English articles

The keyword time-zone map helped to grasp the evolutionary trajectory of related hotspots. As shown in Supplementary Figure S2, the eco-environment research in the YRB from WoS could be divided into three phases.

The first item, the early stage of eco-environment research in YRB (1992–2000), is shown in Supplementary Figure S2a. Clustering hotspots included paleovegetation, the Chinese Loess plateau, evapotranspiration, balance and deposit. The overall number of articles at this stage was relatively low. Until 1998, the annual number of articles published from WoS did not exceed 10. Researchers mainly focused on the relationship between water and sediment in the Loess Plateau. For example, Wang et al. (1999) [37] and Ding et al. (1999) [38] analyzed the effect of desertification on water deficit based on the land water deficit model.

The second item, the slow growth stage of eco-environment research in YRB (2001–2010), is shown in Supplementary Figure S2b,c. The clustering themes included magnetic susceptibility, soil erosion, red clay, and water-use efficiency as well as the Yellow River, Loess Plateau, Chinese Loess Plateau, and YRB. Researchers focused on the relationship between soil erosion and water-use efficiency in the Yellow River and YRB. For example, soil erosion and nutrient losses were monitored, and the runoffs were very sensitive to climate change [39,40]. Additionally, magnetostratigraphy of Late Tertiary sediments and iron geochemistry in the Chinese Loess Plateau were analyzed [41,42].

The third item, the rapid growth period of eco-environment research in YRB (2011–2021), is shown in Supplementary Figure S2d–f. The clustering themes included soil organic carbon, heavy metals, climate change, water-use efficiency, ecological restoration, temporal stability, microstructure, microbial community, as well as the Yellow River, Chinese loess plateau, and Yellow River estuary. During this period, some researchers focused on the relationship between soil organic carbon and water-use efficiency in the semi-arid Loess Plateau, the Loess Plateau, and small watershed areas, and discussed the response of soil moisture, climate change and human activities to vegetation restoration over time [4,43,44,45]. Additionally, some focused on the response of soil fertility, soil respiration and microbial communities to returning farmland to forests and vegetation restoration models in the Loess Plateau and the Loess Plateau mining areas, with more specific and microscopic research contents [46,47,48]. The levels, sources and environmental risks of soil heavy metals in the YRB and the Yellow River Delta also attracted wide attention in this period [49].

- Second bullet; Chinese articles

As shown in Supplementary Figure S3, the eco-environment research in the YRB from CNKI could be divided into the following three phases.

The first item, the early stage of eco-environment research in YRB (1992–2000), is shown in Supplementary Figure S3a. The hot issues mainly included: grain production, yield effect, afforestation, loess, water harvesting agriculture, animal resources and other issues in the Loess Plateau; water resources, soil moisture, paleomonsoon, Late Pleistocene, flood control, water conservancy hub, Sanmenxia, sediment, and other issues in the Yellow River; Yellow River downstream irrigation, water and sand changes, development and utilization, climatic conditions, breakage, impact, river realignment, water and sand combination and other related issues in the lower Yellow River; soil erosion, small watersheds, human activities, protection measures, loess hills, sand production, volcanic flooding period and other related issues in the Yellow River middle reaches; soil conservation, comprehensive management, water to sand, soil resources, ecological benefits, return of farmland to forests, western development and others in the Yellow River Basin; the problems of sand reduction, economic benefit, water scheduling, human factors, water pollution, etc. in the upper reaches of the Yellow River; the problems of the relationship between ecological environment and climate change, development measures, resource utilization, etc.

The second item was the stage of 2001–2010 (Supplementary Figure S3b,c). It focused on the Loess Plateau, Yellow River, Yellow River Basin, lower Yellow River, middle Yellow River, and soil erosion, ecological environment and forestry ecological construction from 2001 to 2005; and focused on the Loess Plateau, Yellow River, Yellow River Basin, Yellow River Estuary and land use, climate change, wetland and vegetation restoration from 2006 to 2010. In the Loess Plateau, it focused on soil erosion, soil moisture to soil humidity, ecological water demand, soil characteristics and other thematic research; In the Yellow River, it focused on water resources, plantation forests, soil nutrients, water quality evaluation, soil conservation, Ningmeng River section, development model, carbon storage and other issues; in the Yellow River Basin, it focused on dynamic monitoring, human activities, management, ecological benefits, load carrying ability, South–North Water Diversion, and other commissioned research; in the lower reaches of the Yellow River, it focused on ecological reconstruction, ecological water use, river-realignment-related trend analysis and statistics analysis; in the middle reaches of the Yellow River, it focused on the Fenwei Plain, Loess Hills and the surrounding areas in terms of governance patterns, ecological construction, Yellow River flooding, land use, ecological water demand; in the Yellow River estuary, it focused on the water environment, organic matter, communities, vegetation succession and other related numerical modeling, coherent analysis; in research hotspots, it mainly focused on soil erosion, ecological environment, forestry ecological construction, land use, climate change, wetlands and vegetation restoration, etc.

The third item was the stage of 2011–2021 (Supplementary Figure S3d–f). It focused on the Loess Plateau, Yellow River, Yellow River Basin, and climate change, soil, wetland, and land use from 2011 to 2015; and focused on the Loess Plateau, Yellow River Basin, the Yellow River, Yellow River Estuary, and land use, yield and water quality from 2016 to 2021. In the Loess Plateau, it focused on the dry soil layer, soil properties, carbon storage, carbon density, erosion, and total nitrogen in the Loess hills; in the Yellow River, it focused on heavy metals, suspended matter, water balance, incoming sand coefficient, human activities, siltation, climate factors, and watershed ecology in the semi-arid region; in the Yellow River Basin, it focused on the main stream of the Yellow River, the source area of the Yellow River, soils, organic carbon, saline sedimentary context, topography and geomorphology, sensitivity, spatial and temporal patterns, ecological restoration and other issues; in the Yellow River estuary, it mainly explored environmental factors, ecological environment, ecological management, ecological protection-related issues and drivers; it focused on hot topics for climate change, soil, wetlands, land use, yield and water quality.

#### 3.2.4. Research Frontier Analysis

**Figure 6:**
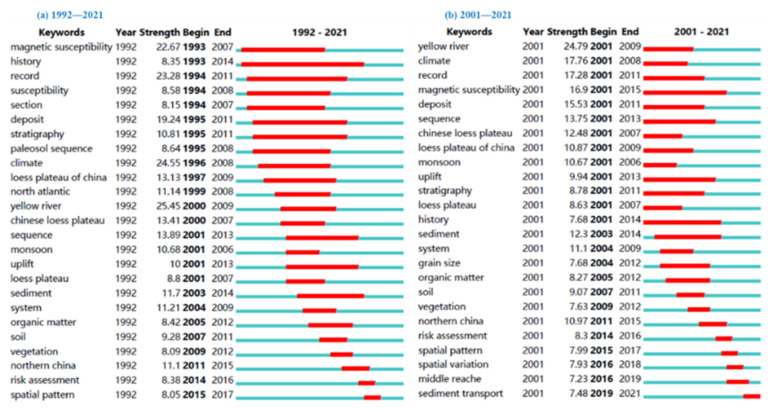
Top 25 keywords with the strongest citation bursts from WoS.

**Figure 7:**
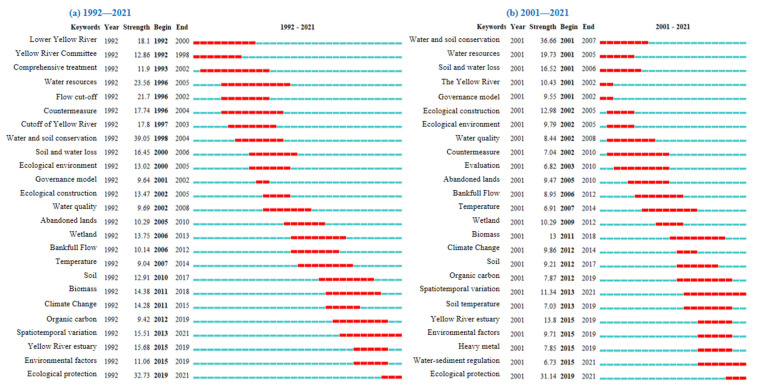
Top 25 keywords with the strongest citation bursts from CNKI.

The keyword emergence mapping by CiteSpace software could provide a good option for identifying hotspots and emerging frontiers in the research field. By identifying and tracking the frontier hotspots, it was helpful to understanding the latest evolutions of research field and finding out the problems requiring further research. On this basis, the keyword emergent mapping was analyzed for two time periods, 1992–2021 and 2001–2021 from Wos (Figure 6) and CNKI (Figure 7).

- First bullet; English articles

From the WoS database, 25 emergent words were obtained (Figure 6). It was found that from 1992–2021, the emergent words were magnetic susceptibility, history, record, susceptibility, section, deposit, stratigraphy, paleosol sequence, climate, Loess Plateau of China, North Atlantic, yellow river, Chinese Loess Plateau, sequence, monsoon, uplift, loess plateau, sediment, system, organic matter, soil, vegetation, northern China, risk assessment, spatial pattern; from 2001–2021, the emergent words were Yellow River, climate, record, magnetic susceptibility, deposit, sequence, Chinese Loess Plateau, Loess Plateau of China, monsoon, uplift, stratigraphy, Loess Plateau, history, sediment, system, grain size, organic matter, soil, vegetation, northern China, risk assessment, spatial pattern, spatial variation, middle reaches, sediment transport.

- Second bullet; Chinese articles

From the CNKI database, 25 emergent words were obtained (Figure 7). Comparing the analysis, it was found that from 1992–2000, the emergent words were lower Yellow River, Yellow River Commission, comprehensive management, water resources, cutoff, countermeasures, Yellow River cutoff, soil conservation, and ecological environment, mainly focusing on countermeasures and measures related to Yellow River cutoff; from 2001–2010, the emergent words were governance mode, ecological construction, water quality, abandoned lands, soil conservation, water resources, soil erosion, Yellow River, ecological environment, water quality, countermeasures, and evaluation, mainly focusing on the governance model, countermeasures and evaluation of the Yellow River; from 2011–2018, the emergent words were wetland, bankfull flow, temperature, soil, biomass, climate change, organic carbon, spatiotemporal variation, Yellow River estuary, environmental factors, soil temperature, and heavy metals, mainly focusing on the spatial and temporal changes characteristics of temperature, biomass, organic carbon and related climate change and environmental factors of the Yellow River estuary and wetlands; from 2019–2021, the emergent words were ecological protection and water and sediment regulation. With the continuous development of the YRB governance technology, the problems related to water, soil, resources and environment of YRB have been effectively managed and standardized. The ecological protection and high-quality development in the YRB has become the focus of current scholars.

## 4. Discussion

First, further research trends change as policies change. For example, from 1992 to 2000, the related papers mainly focused the problems of “water–sand–environment”. During this research period, “Guangming Daily” published the report of “Research on the disconnection of the Yellow River”. It proposed the “forum of young scientists” that was held by the China Association for Science and Technology. Frontiers of the environment, water resources, geography, and sedimentation cross-cutting science were discussed. On this basis, it was proposed to carry out a “study on the ecological environmental impact of the Yellow River breakwater on the watershed system”. From 2001 to 2010, the “Eleventh Five-Year Plan” emphasized improving the environmental quality of key watersheds and key areas and strengthening the prevention and control of water pollution along the middle and upper reaches of the Yellow River and South-to-North Water Transfer Project. By 2016, the management of the Yellow River had gone through 70 years (The people’s cause of Yellow River management under the leadership of the Communist Party of China started in 1946), and the development and management of the Yellow River has achieved remarkable results. From 2020 to 2021, with the ecological protection and high-quality development of the Yellow River Basin emerging as a national strategy, the related papers of this period focused on the YRB, Loess Plateau, Yellow River, Lower Yellow River and related issues such as community structure precipitation, land use and land remediation.

Second, research hotspot analysis has promoted the faster development of eco-environment research in the YRB. With the ecological protection and high-quality development of the YRB emerging as a national strategy, the number of related articles in the future should also show a rapid growth trend (Figure 2), and the research topics should be more extensive. According to the hotspot analysis, it mainly focused on two aspects. One was the relationship between climate change, human activities and ecological quality in the YRB, especially focusing on the quantitative evaluation and systematic analysis of the relevant influencing factors. The second was the ecological health and human well-being of the YRB, which promote the coupling and coordinated development of economy, ecology, environment and well-being, and promote its sustainable and high-quality development. Future research on eco-environment in the YRB should also be focused on local high-quality development and ecological protection. The ecological restoration, climate change, land use, community structure and sustainable development in the YRB, as well as the Loess Plateau and the Yellow river estuary should be of concern [9,50].

Third, the overall research on the eco-environment in the YRB has changed from quantity to quality, from utilization to management, from macro to micro, and from construction to high-quality development. The hot topics of water research in the YRB have changed from water quantity to water quality, from early concern about soil conservation and soil erosion to recent concern about water quality changes; the focus of land research has changed from utilization to management, from early concern about land use, wetland and soil to recent concern about land remediation; the focus of vegetation research has changed from macro to micro, from early concern about vegetation restoration to recent concern about community. Based on hotspot analysis, the hot topic of ecological protection research has changed from ecological construction to high-quality development, from an early focus on forestry ecological construction, ecological environment and climate change to a recent focus on high-quality development of the three-living spaces and coupling and coordination.

Fourth, the present study performed bibliometric analysis of the eco-environment research in the YRB over the past thirty years. This work analyzed its characteristics and hotspots, which could explore its progress and frontiers. Meanwhile, this work also has some limitations that could be improved in the future. For example, this work mainly focused on publications in English and Chinese, and only considered the WoS and CNKI databases, which caused inevitable defects (that is, the data used may be less than all the relevant data). In addition, a bibliometric analysis involves all aspects. This paper considered the characteristics research hotspots of publications. For example, citations were not discussed in this paper. Future work should consider these deficiencies.

## 5. Conclusions

By analyzing the characteristics and research hotspots of eco-environment research in the YRB from 1992 to 2021, the following conclusions were mainly drawn, which provide reference for further promoting ecological environment research in the YRB.

First, in terms of the total number of articles, the overall research on eco-environment in the YRB showed an upward trend from WoS and a fluctuating upward trend (up-down-up) from CNKI, especially since the ecological protection and high-quality development of the YRB have emerged as a national strategy and since the research related to the eco-environment in the YRB has attracted more attention from scholars and governments. Research on the eco-environment in the YRB has been multidisciplinary; the disciplines and journals with higher publications were mainly related to the YRB or to its resources, ecology and environment; the research institutions with a greater number of publication were mainly concentrated in Beijing or around the YRB. In terms of discipline development, it was necessary to further strengthen the multi-disciplinary intersection; in terms of cooperation and exchange, it was necessary to further promote multi-faceted cooperation among universities, research institutions, enterprises and related journals and authors, so as to achieve complementary advantages and promote deep integration of industry, academia and research. In terms of the evolution of the study area, it was mainly focused on the Loess Plateau, the Yellow River and the Yellow River Basin. The early stage of the study focused on the large areas of the lower reaches of the Yellow River, the middle reaches of the Yellow River and the upper reaches of the Yellow River; in the middle and late stages of the study, it focused on the mouth of the Yellow River, and the recent study focused on the source area of the Yellow River.

Second, the hot topics of research mainly included soil conservation, soil erosion, vegetation restoration, forestry ecological construction, ecological environment, water quality, land use, climate change, community structure, three-life space, and coupling and coordination. From the perspective of the theme of evolution, the “water–soil–vegetation–ecological protection” series of problems have been of general concern. The hot topic of ecological protection research has changed from ecological construction to high-quality development, from an early focus on forestry ecological construction, ecological environment and climate change to a recent focus on high-quality development of the three-living spaces and coupling and coordination. From the research frontier, water, soil, resources, and environment in the YRB have received general attention, and the related management has been remarkably effective. How to achieve ecological protection and high-quality development in the YRB is the most important issue currently being faced, and is also a focal topic for research purposes. In future research, it will be necessary to conduct in-depth research on water, soil, ecological environment and local high-quality development in small regions and small watersheds.

## Figures and Tables

**Table 1:** Top 10 institutions for eco-environment research in YRB.

WoS	Institutions	Country	Number	Ratio (%)
1	Chinese Academy of Sciences	China	2161	16.2
2	Northwest A&F University	China	1086	8.1
3	Ministry Water Resources	China	563	4.2
4	Beijing Normal University	China	482	3.6
5	Lanzhou University	China	359	2.7
6	Xian University of Technology	China	155	1.2
7	Ocean University of China	China	153	1.2
8	Shaanxi Normal University	China	131	1.0
9	Beijing Forestry University	China	128	1.0
10	China Institute of Water Resources and Hydropower Research	China	121	0.9
**CNKI**	**Institutions**	**Province**	**Number**	**Ratio (%)**
1	Northwest A&F University	Shaanxi	1643	5.3
2	Ministry Water Resources	Shaanxi	1088	3.5
3	Institute of Geographic Sciences Natural Resources Research CAS	Beijing	497	1.6
4	Yellow River Institute of Hydraulic Research, Yellow River Conservancy Commission	Henan	448	1.4
5	Beijing Normal University	Beijing	413	1.3
6	University of Chinese Academy of Science CAS	Beijing	404	1.3
7	Lanzhou University	Gansu	397	1.3
8	Shaanxi Normal University	Shaanxi	309	1.0
9	Beijing Forestry University	Beijing	254	0.8
10	Ocean University of China	Shandong	231	0.7

**Table 2:** Top 10 productive journals for the research on eco-environment in the YRB.

WoS	Journals	Number	Ratio (%)	2021 IF	Country
1	Catena	238	4.47	6.367	Germany
2	Science of The Total Environment	208	3.91	10.753	Netherlands
3	Water	124	2.33	3.530	Switzerland
4	Journal of Hydrology	113	2.12	6.708	Netherlands
5	Environmental Earth Sciences	92	1.73	3.119	Germany
6	Agricultural Water Management	89	1.67	6.611	Netherlands
7	Land Degradation & Development	85	1.60	4.377	England
8	Hydrological Processes	84	1.58	3.784	England
9	Sustainability	82	1.54	3.889	Switzerland
10	PLoS One	79	1.48	3.752	USA
**CNKI**	**Journals**	**Number**	**Ratio (%)**	**2021 IF**	**Province**
1	Yellow River	1593	13.2	1.067	Henan
2	Bulletin of Soil and Water Conservation	448	3.71	1.894	Shaanxi
3	Acta Ecologica Sinica	444	3.68	4.733	Beijing
4	Research of Soil and Water Conservation	387	3.21	2.987	Shaanxi
5	Journal of Soil and Water Conservation	359	2.98	3.198	Shaanxi
6	Science of Soil and Water Conservation	233	1.93	1.922	Beijing
7	Agricultural Research in the Arid Areas	229	1.90	1.706	Shaanxi
8	Chinese Journal of Applied Ecology	208	1.72	3.893	Liaoning
9	Journal of Arid Land Resources and Environment	193	1.60	3.187	Inner Mongolia
10	Journal of Natural Resources	191	1.58	6.098	Beijing

## Data Availability

Not applicable.

## Supplementary Materials

The following supporting information can be downloaded at: https://www.mdpi.com/1660-4601/19/19/11986/s1. Figure S1: Keyword co-occurrence network map in the eco-environment research in YRB from CNKI (CSCD, CSSCI, and Core). Figure S2: Phased topic evolution map of papers in the eco-environment research in YRB from WoS platform. Figure S3: Phased topic evolution map of papers in the eco-environment research in YRB from CNKI (CSCD, CSSCI, and Core). Table S1: Highly cited scientific papers in the eco-environment research in the Yellow River Basin from WoS platform. Table S2: TOP 20 Highly cited scientific papers in the eco-environment research in the Yellow River Basin from CNKI (in English).

## References

[1] Nie J.S., Stevens T., Rittner M., Stockli D., Garzanti E., Limonta M., Bird A., Andò S., Vermeesch P., Saylor J. (2015). Loess Plateau storage of Northeastern Tibetan Plateau-derived Yellow River sediment. Nat. Commun..

[2] Wang S., Fu B.J., Piao S.L., Lü Y.H., Ciais P., Feng X.M., Wang Y.F. (2016). Reduced sediment transport in the Yellow River due to anthropogenic changes. Nat. Geosci..

[3] The Communist Party of China Central Committee (CPCC) and the State Council (2021). Outline Document on the Ecological Protection and High-Quality Development of the Yellow River Basin. http://www.gov.cn/zhengce/2021-10/08/content_5641438.htm.

[4] Zheng K., Wei J.Z., Pei J.Y., Cheng H., Zhang X.L., Huang F.Q., Li F.M., Ye J.S. (2019). Impacts of climate change and human activities on grassland vegetation variation in the Chinese Loess Plateau. Sci. Total. Environ..

[5] Han Z.M., Huang Q., Huang S.Z., Leng G.Y., Bai Q.J., Liang H., Wang L., Zhao J., Fang W. (2021). Spatial-temporal dynamics of agricultural drought in the Loess Plateau under a changing environment: Characteristics and potential influencing factors. Agr. Water Manag..

[6] Zhang Y.L., Zhao Z.Y., Fu B.J., Ma R.M., Yang Y.Y., Lü Y.H., Wu X. (2022). Identifying ecological security patterns based on the supply, demand and sensitivity of ecosystem service: A case study in the Yellow River Basin, China. J. Environ. Manag..

[7] Chen Y.P., Fu B.J., Zhao Y., Wang K.B., Zhao M.M., Ma J.F., Wu J.H., Xu C., Liu W.G., Wang H. (2020). Sustainable development in the Yellow River Basin: Issues and strategies. J. Clean. Prod..

[8] Fang L.L., Wang L.C., Chen W.X., Sun J., Cao Q., Wang S.Q., Wang L.Z. (2021). Identifying the impacts of natural and human factors on ecosystem service in the Yangtze and Yellow River Basins. J. Clean. Prod..

[9] Shi S.Y., Yu Y.J., Wang F., Wang P., Zhang Y.C., Jin K. (2021). Quantitative contributions of climate change and human activities to vegetation changes over multiple time scales on the Loess Plateau. Sci. Total. Environ..

[10] Feng K.S., Siu Y.L., Guan D.B., Hubacek K. (2012). Assessing regional virtual water flows and water footprints in the Yellow River Basin, China: A consumption based approach. Appl. Geogr..

[11] Jian S.Q., Zhao C.Y., Fang S.M., Yu K. (2015). Effects of different vegetation restoration on soil water storage and water balance in the Chinese Loess Plateau. Agr. Forest. Meteorol..

[12] Chen J., Gao Y.Y., Qian H., Jia H., Zhang Q.Y. (2021). Insights into water sustainability from a grey water footprint perspective in an irrigated region of the Yellow River Basin. J. Clean. Prod..

[13] Bai J.H., Xiao R., Zhang K.J., Gao H.F. (2012). Arsenic and heavy metal pollution in wetland soils from tidal freshwater and salt marshes before and after the flow-sediment regulation regime in the Yellow River Delta, China. J. Hydrol..

[14] He X.D., Li P.Y. (2020). Surface water pollution in the middle Chinese Loess Plateau with special focus on hexavalent chromium (Cr6+): Occurrence, sources and health risks. Expos. Health..

[15] Zhang Z.H., Zhang G.X., Su B. (2022). The spatial impacts of air pollution and socio-economic status on public health: Empirical evidence from China. Socio. Econ. Plan. Sci..

[16] Zhang Z.H., Zhang G.X., Li L.L. (2022). The spatial impact of atmospheric environmental policy on public health based on the mediation effect of air pollution in China. Environ. Sci. Pollut. Res..

[17] Zhang Y.S., Lu X., Liu B.Y., Wu D.T., Fu G., Zhao Y.T., Sun P.L. (2021). Spatial relationships between ecosystem services and socioecological drivers across a large-scale region: A case study in the Yellow River Basin. Sci. Total. Environ..

[18] Geng W.L., Li Y.Y., Zhang P.Y., Yang D., Jing W.L., Rong T.Q. (2022). Analyzing spatio-temporal changes and trade-offs/synergies among ecosystem services in the Yellow River Basin, China. Ecol. Indic..

[19] He X.D., Wu J.H., He S. (2018). Hydrochemical characteristics and quality evaluation of groundwater in terms of health risks in Luohe aquifer in Wuqi County of the Chinese Loess Plateau, northwest China. Hum. Ecol. Risk. Assess..

[20] Zhang P.Y., Qin C.Z., Hong X., Kang G.H., Qin M.Z., Yang D., Pang B., Li Y.Y., He J.J., Dick R.P. (2018). Risk assessment and source analysis of soil heavy metal pollution from lower reaches of Yellow River irrigation in China. Sci. Total. Environ..

[21] Li P.Y., He X.D., Guo W.Y. (2018). Spatial groundwater quality and potential health risks due to nitrate ingestion through drinking water: A case study in Yan’an City on the Loess Plateau of northwest China. Hum. Ecol. Risk. Assess..

[22] Xiao J., Wang L.Q., Deng L., Jin Z.D. (2019). Characteristics, sources, water quality and health risk assessment of trace elements in river water and well water in the Chinese Loess Plateau. Sci. Total. Environ..

[23] Wohlfart C., Kuenzer C., Chen C., Liu G.H. (2016). Social–ecological challenges in the Yellow River basin (China): A review. Environ. Earth. Sci..

[24] Lu H.F., Qi X.B., Qiao D.M. (2020). Using bibliometrics to analyze research on irrigation and drainage in the Yellow River Basin. J. Irrig. Drain..

[25] He Z.H., Gong K.Y., Zhang Z.L., Dong W.B., Feng H., Yu Q., He J.Q. (2022). What is the past, present, and future of scientific research on the Yellow River Basin?—A bibliometric analysis. Agr. Water. Manag..

[26] Liu L.N., Qu J.S., Maraseni T.N., Niu Y.B., Zeng J.J., Zhang L.H., Xu L. (2020). Household CO_2_ Emissions: Current Status and Future Perspectives. Int. J. Environ. Res. Public Health.

[27] Zeng J.J., Qu J.S., Ma H.Q., Gou X.H. (2021). Characteristics and Trends of household carbon emissions research from 1993 to 2019: A bibliometric analysis and its implications. J. Clean. Prod..

[28] Chen C.M. (2006). CiteSpace II: Detecting and visualizing emerging trends and transient patterns in scientific literature. J. Am. Soc. Inf. Sci. Technol..

[29] (2022). Sourceforge. https://sourceforge.net/projects/citespace/.

[30] (2022). VOSviewer.

[31] Van Eck N.J., Waltman L. (2010). Software survey: VOSviewer, a computer program for bibliometric mapping. Scientometrics.

[32] Han B., Chen R.X., Tian S.M., Cao Y.T., Wang X. Trend analysis of research on the ecological protection of Yellow River Basin on Bibliometrics. Proceedings of the 2021 Annual Science and Technology Conference of the Chinese Society of Environmental Science.

[33] Feng X.M., Fu B.J., Piao S.L., Wang S., Ciais P., Zeng Z.Z., Lü Y.H., Zeng Y., Jiang X.H., Wu B.F. (2016). Revegetation in China’s Loess Plateau is approaching sustainable water resource limits. Nat. Clim. Chang..

[34] Fu B.J., Ma K.M., Zhou H.F., Chen L.X. (1998). Influence of land use structure on soil nutrient distribution in loess hilly region. Chin. Sci. Bull..

[35] Fu B.J., Chen L.D., Ma K.M. (1999). The effect of land use change on the regional environment in the YangJuanGou catchment in the loess plateau of China. Acta Geogr. Sin..

[36] Fu B.J., Qiu Y., Wang J., Chen L.X. (2002). Effect simulations of land use change on the runoff and erosion for a Gully Catchment of the Loess Plateau, China. Acta Geogr. Sin..

[37] Wang Q.X., Takahashi H. (1999). A land surface water deficit model for an arid and semiarid region: Impact of desertification on the water deficit status in the Loess Plateau, China. J. Clim..

[38] Ding Z.L., Sun J.M., Rutter N.W., Rokosh D., Liu T. (1999). Changes in sand content of loess deposits along a north-south transect of the Chinese Loess Plateau and the implications for desert variations. Quat. Res..

[39] Zhang F.L. (2005). Effects of accelerated soil erosion on soil nutrient loss after deforestation on the Loess Plateau. Pedosphere.

[40] Guo S.L., Chen H., Zhang H.G., Xiong L.H., Liu P., Pang B., Wang G.Q., Wang Y.Z. (2005). A semi-distributed monthly water balance model and its application in a climate change impact study in the middle and lower Yellow River basin. Water Int..

[41] Song Y.G., Fang X.M., Masayuki T., Naoto I., Li J.J., An Z.S. (2001). Magnetostratigraphy of Late Tertiary sediments from the Chinese Loess Plateau and its paleoclimatic significance. Chinese. Sci. Bull..

[42] Ding Z.L., Yang S.L., Sun J.M., Liu T.S. (2001). Iron geochemistry of loess and red clay deposits in the Chinese Loess Plateau and implications for long-term Asian monsoon evolution in the last 7.0 Ma. Earth. Planet. Sc. Lett..

[43] Zhao X., Wu P., Gao X., Persaud N. (2012). Soil quality indicators in relation to land use and topography in a small catchment on the loess plateau of China. Land. Degrad. Dev..

[44] Li R., Hou X.Q., Jia Z.K., Han Q.F., Ren X.L., Yang B.P. (2013). Effects on soil temperature, moisture, and maize yield of cultivation with ridge and furrow mulching in the rainfed area of the Loess Plateau, China. Agr. Water. Manag..

[45] Yang L., Wei W., Chen L.D., Chen W.L., Wang J.L. (2014). Response of temporal variation of soil moisture to vegetation restoration in semi-arid Loess Plateau, China. Caten..

[46] Zhang L., Lv J.P. (2020). Metagenomic analysis of microbial community and function reveals the response of soil respiration to the conversion of cropland to plantations in the Loess Plateau of China. Glob. Ecol. Conserv..

[47] Han J.Q., Dong Y.Y., Zhang M. (2021). Chemical fertilizer reduction with organic fertilizer effectively improve soil fertility and microbial community from newly cultivated land in the Loess Plateau of China. Appl. Soil. Ecol..

[48] Zhao J., Ma J., Yang Y.J., Yu H.C., Zhang S.L., Chen F. (2021). Response of soil microbial community to vegetation reconstruction modes in Mining Areas of the Loess Plateau, China. Front. Microbiol..

[49] Zhang G.L., Bai J.H., Zhao Q.Q., Lu Q.Q., Jia J., Wen X.J. (2016). Heavy metals in wetland soils along a wetland-forming chronosequence in the Yellow River Delta of China: Levels, sources and toxic risks. Ecol. Indic..

[50] Dong X.B., Wang X.W., Wei H.J., Fu B.J., Wang J.J., Uriarte-Ruiz M. (2021). Trade-offs between local farmers’ demand for ecosystem services and ecological restoration of the Loess Plateau, China. Ecosyst. Serv..

