# Supplementary material for: Review on Eco-Environment Research in the Yellow River Basin: A Bibliometric Perspective
Source: Int J Environ Res Public Health. 2022 Sep 22;19(19):11986. doi: 10.3390/ijerph191911986 (PMC9565096; doi:10.3390/ijerph191911986)
Supplement: Supplementary file 1 [file ijerph-19-11986-s001.zip › ijerph-1917827-Supplementary Materials.pdf]

Supplementary Figures

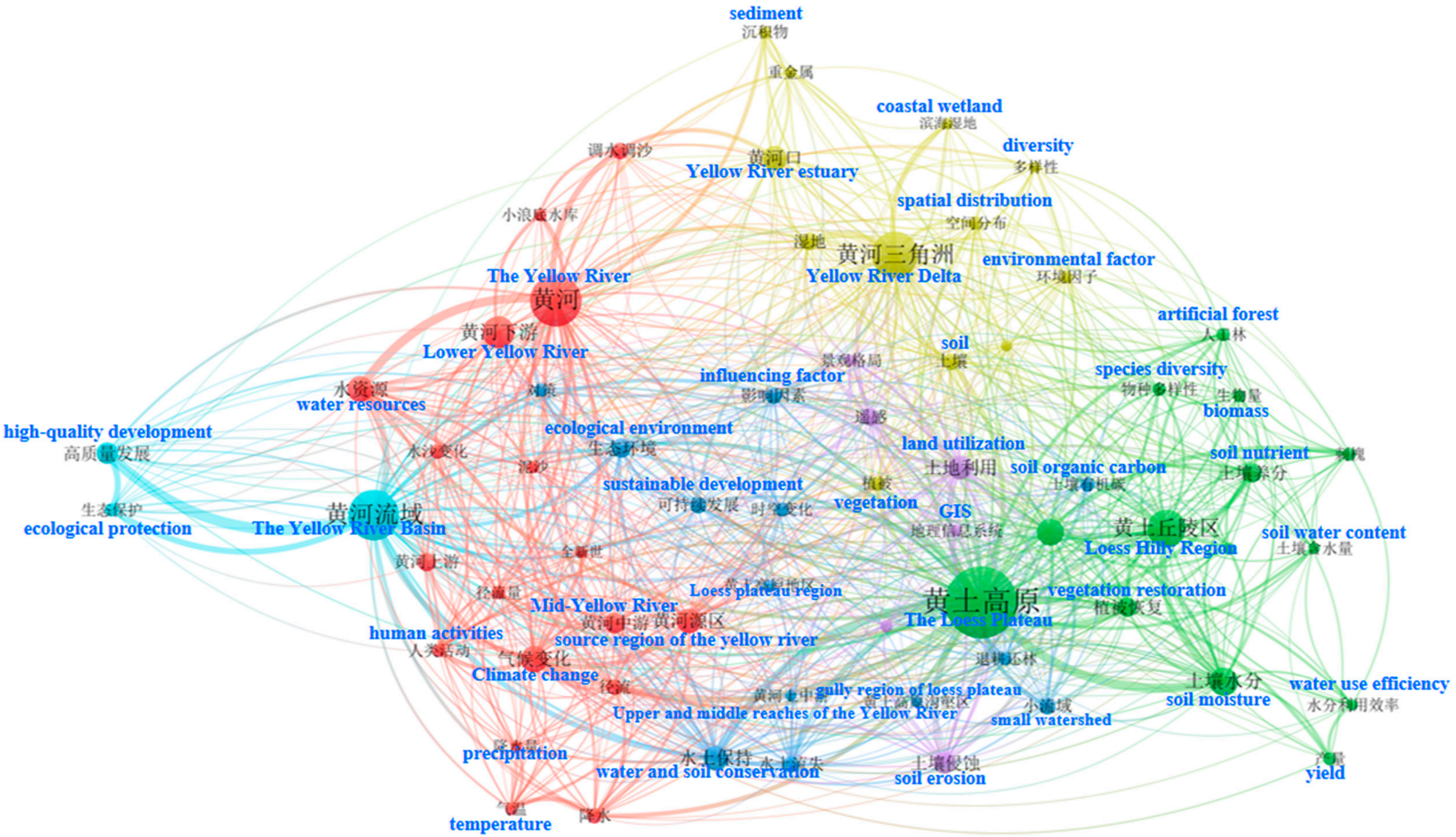

Supplementary Figure S1. Keyword co-occurrence network map in the eco-environment research in YRB from CNKI (CSCD, CSSCI, and Core).

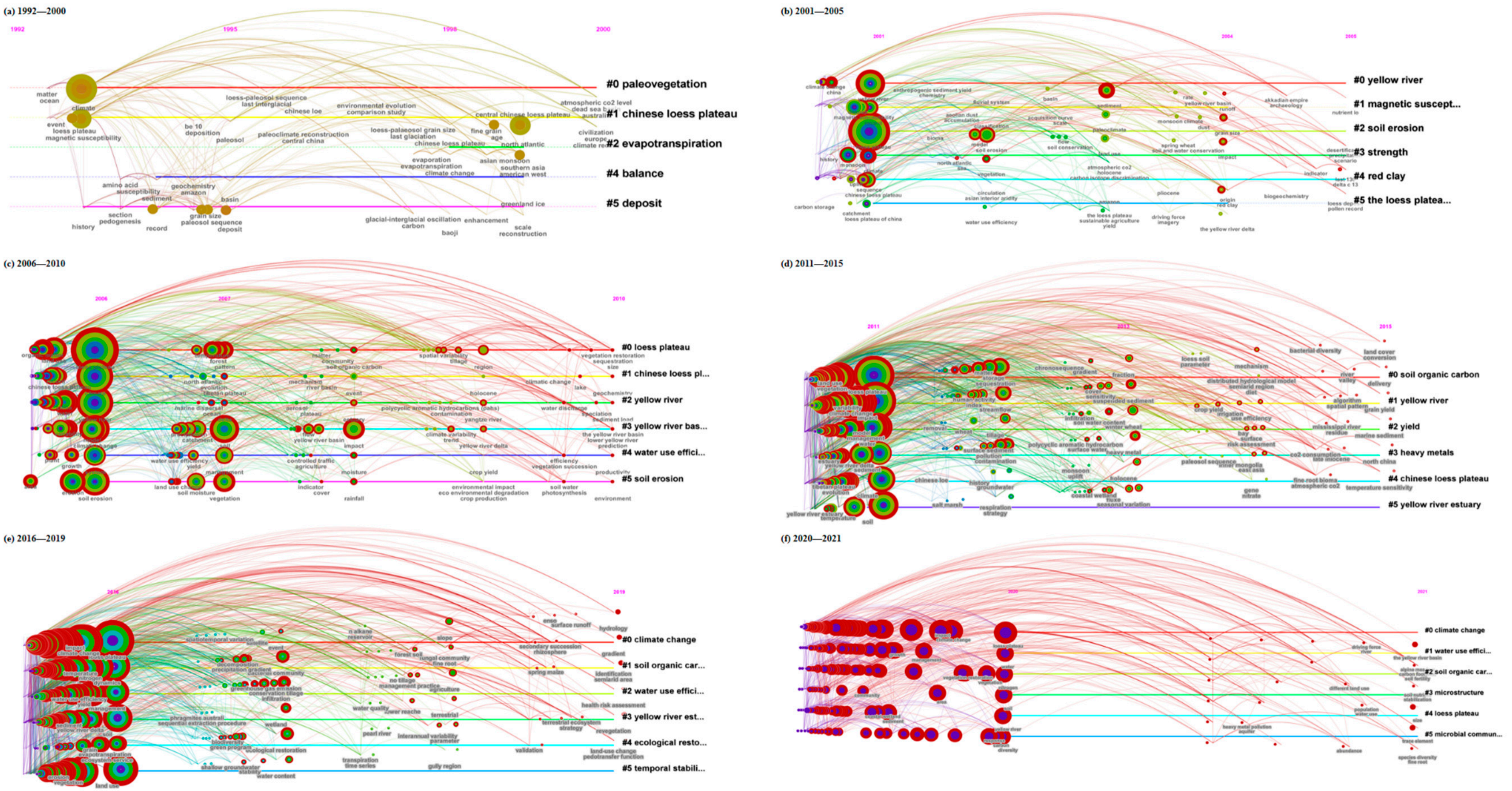

Supplementary Figure S2. Phased topic evolution map of papers in the eco-environment research in YRB from WoS platform.

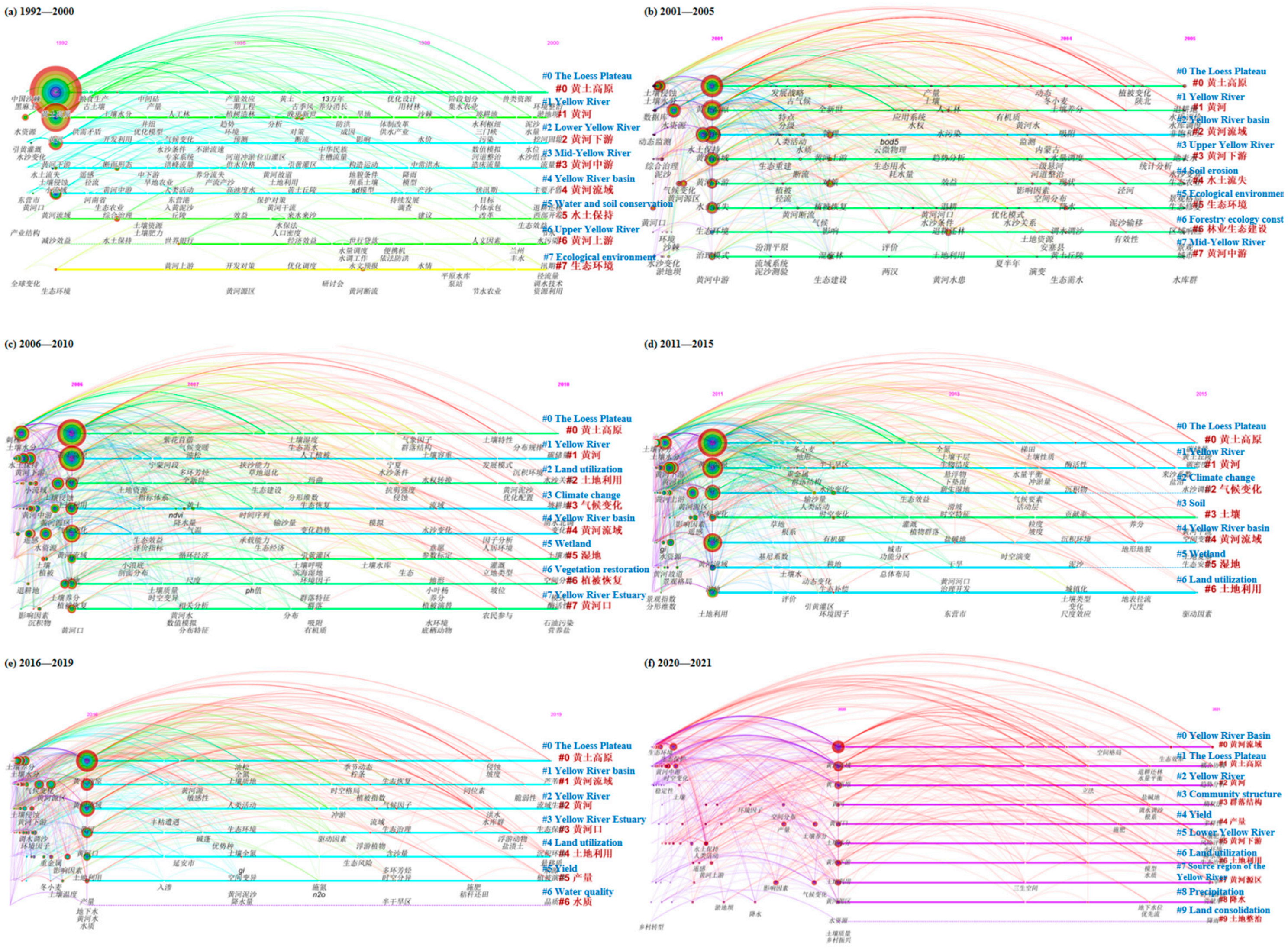
(a) 1992—2000
#0 The Loess Plateau
#0 黄土高原
#1 Yellow River
#1 黄河
#2 Lower Yellow River
#2 黄河下游
#3 Mid-Yellow River
#3 黄河中游
#4 Yellow River basin
#4 黄河流域
#5 Water and soil conservation
#5 水土保持
#6 Upper Yellow River
#6 黄河上游
#7 Ecological environment
#7 生态环境
(b) 2001—2005
#0 The Loess Plateau
#0 黄土高原
#1 Yellow River
#1 黄河
#2 Yellow River basin
#2 黄河流域
#3 Upper Yellow River
#3 黄河下游
#4 Soil erosion
#4 水土流失
#5 Ecological environmen
#5 生态环境
#6 Forestry ecology const
#6 林业生态建设
#7 Mid-Yellow River
#7 黄河中游
(c) 2006—2010
#0 The Loess Plateau
#0 黄土高原
#1 Yellow River
#1 黄河
#2 Land utilization
#2 土地利用
#3 Climate change
#3 气候变化
#4 Yellow River basin
#4 黄河流域
#5 Wetland
#5 湿地
#6 Vegetation restoration
#6 植被恢复
#7 Yellow River Estuary
#7 黄河口
(d) 2011—2015
#0 The Loess Plateau
#0 黄土高原
#1 Yellow River
#1 黄河
#2 Climate change
#2 气候变化
#3 Soil
#3 土壤
#4 Yellow River basin
#4 黄河流域
#5 Wetland
#5 湿地
#6 Land utilization
#6 土地利用
(e) 2016—2019
#0 The Loess Plateau
#0 黄土高原
#1 Yellow River basin
#1 黄河流域
#2 Yellow River
#2 黄河
#3 Yellow River Estuary
#3 黄河口
#4 Land utilization
#4 土地利用
#5 Yield
#5 产量
#6 Water quality
#6 水质
(f) 2020—2021
#0 Yellow River Basin
#0 黄河流域
#1 The Loess Plateau
#1 黄土高原
#2 Yellow River
#2 黄河
#3 Community structure
#3 群落结构
#4 Yield
#4 产量
#5 Lower Yellow River
#5 黄河下游
#6 Land utilization
#6 土地利用
#7 Source region of the Yellow River
#7 黄河源区
#8 Precipitation
#8 降水
#9 Land consolidation
#9 土地整治

Supplementary Figure S3. Phased topic evolution map of papers in the eco-environment research in YRB from CNKI (CSCD, CSSCI, and Core).

Supplementary Tables

Supplementary Table S1. Highly cited scientific papers in the eco-environment research in the Yellow River Basin from WoS platform

NO.	Title	First Author	Journal	Year	Times
1	Revegetation in China's Loess Plateau is approaching sustainable water resource limits	Feng, Xiaoming	Nat. Clim. Chang.	2016	673
2	Reduced sediment transport in the Yellow River due to anthropogenic changes	Wang, Shuai	Nat. Geosci.	2016	565
3	Soil erosion, conservation, and eco-environment changes in the loess plateau of china	Zhao, Guangju	Land Degrad. Dev.	2013	553
4	A Policy-Driven Large Scale Ecological Restoration: Quantifying Ecosystem Services Changes in the Loess Plateau of China	Lu, Yihe	PLoS One	2012	346
5	Soil moisture decline due to afforestation across the Loess Plateau, China	Jia, Xiaoxu	J. Hydrol.	2017	264
6	Quantifying the impacts of climate change and ecological restoration on streamflow changes based on a Budyko hydrological model in China's Loess Plateau	Liang, Wei	Water Resour. Res.	2015	262
7	Soil bacterial community dynamics reflect changes in plant community and soil properties during the secondary succession of abandoned farmland in the Loess Plateau	Zhang, Chao	Soil Biol. Biochem.	2016	251
8	Occurrence and Health Implication of Fluoride in Groundwater of Loess Aquifer in the Chinese Loess Plateau: A Case Study of Tongchuan, Northwest China	Li, Peiyue	Expo. Health	2019	246
9	Assessment of heavy metals contamination in sediments from three adjacent regions of the Yellow River using metal chemical fractions and multivariate analysis techniques	Ma, Xiaoling	Chemosphere	2016	239
10	Spatiotemporal vegetation cover variations associated with climate change and ecological restoration in the Loess Plateau	Sun, Wenyi	Agric. For. Meteorol.	2015	235
11	Loess Plateau storage of Northeastern Tibetan Plateau-derived Yellow River sediment	Nie, Junsheng	Nat. Commun.	2015	225
12	Quantifying the impact of climate variability and human activities on streamflow in the middle reaches of the Yellow River basin, China	Zhao, Guangju	J. Hydrol.	2014	217
13	Effects on soil temperature, moisture, and maize yield of cultivation with ridge and furrow mulching in the rainfed area of the Loess Plateau, China	Li, Rong	Agric. Water Manage.	2013	212
14	Assessing the effects of land use and topography on soil erosion on the Loess Plateau in China	Sun, Wenyi	Catena	2014	208
15	Assessing regional virtual water flows and water footprints in the Yellow River Basin, China: A consumption based approach	Feng, Kuishuang	Appl. Geogr.	2012	198
16	Arsenic and heavy metal pollution in wetland soils from tidal freshwater and salt marshes before and after the flow-sediment regulation regime in the Yellow River Delta, China	Bai, Junhong	J. Hydrol.	2012	185
17	Characteristics, sources, water quality and health risk assessment of trace elements in river water and well water in the Chinese Loess Plateau	Xiao, Jun	Sci. Total Environ.	2019	179
18	Effects of different vegetation restoration on soil water storage and water balance in the Chinese Loess Plateau	Jian, Shengqi	Agric. For. Meteorol.	2015	176
19	Evolution of the Yellow River Delta and its relationship with runoff and sediment load from 1983 to 2011	Kong, Dongxian	J. Hydrol.	2015	168
20	Changes in extreme temperature and precipitation events in the Loess Plateau (China) during 1960-2013 under global warming	Sun, Wenyi	Atmos. Res.	2016	157

**21**	Spatial groundwater quality and potential health risks due to nitrate ingestion through drinking water: A case study in Yan'an City on the Loess Plateau of northwest China	Li, Peiyue	Hum. Ecol. Risk Assess.	2019	150
**22**	Risk assessment and source analysis of soil heavy metal pollution from lower reaches of Yellow River irrigation in China	Zhang, Pengyan	Sci. Total Environ.	2018	147
**23**	Hydrochemical characteristics and quality evaluation of groundwater in terms of health risks in Luohe aquifer in Wuqi County of the Chinese Loess Plateau, northwest China	He, Xiaodong	Hum. Ecol. Risk Assess.	2019	144
**24**	Response of temporal variation of soil moisture to vegetation restoration in semi-arid Loess Plateau, China	Yang, Lei	Catena	2014	137
**25**	Detecting and attributing vegetation changes on China's Loess Plateau	Li, Jingjing	Agric. For. Meteorol.	2017	127
**26**	Effects of precipitation and restoration vegetation on soil erosion in a semi-arid environment in the Loess Plateau, China	Zhou, Ji	Catena	2016	125
**27**	Use of biochar-compost to improve properties and productivity of the degraded coastal soil in the Yellow River Delta, China	Luo, Xianxiang	J. Soils Sediments	2017	118
**28**	Statistical and multivariate statistical techniques to trace the sources and affecting factors of groundwater pollution in a rapidly growing city on the Chinese Loess Plateau	Wu, Jianhua	Hum. Ecol. Risk Assess.	2020	113
**29**	Ecoenzymatic stoichiometry and microbial nutrient limitation in rhizosphere soil in the arid area of the northern Loess Plateau, China	Cui, Yongxing	Soil Biol. Biochem.	2018	112
**30**	Distribution and characteristics of landslide in Loess Plateau: A case study in Shaanxi province	Zhuang, Jianqi	Eng. Geol.	2018	111
**31**	Comprehensive understanding of groundwater quality for domestic and agricultural purposes in terms of health risks in a coal mine area of the Ordos basin, north of the Chinese Loess Plateau	Wu, Jianhua	Environ. Earth Sci.	2019	110
**32**	Impacts of climate change and human activities on grassland vegetation variation in the Chinese Loess Plateau	Zheng, Kai	Sci. Total Environ.	2019	106
**33**	Surface Water Pollution in the Middle Chinese Loess Plateau with Special Focus on Hexavalent Chromium (Cr6+): Occurrence, Sources and Health Risks	He, Xiaodong	Expo. Health	2020	101
**34**	A fluidized landslide occurred in the Loess Plateau: A study on loess landslide in South Jingyang tableland	Leng, Yanqiu	Eng. Geol.	2018	93
**35**	Distribution of microplastics in surface water of the lower Yellow River near estuary	Han, Mei	Sci. Total Environ.	2020	86
**36**	Assessment of the sustainability of Gymnocypris eckloni habitat under river damming in the source region of the Yellow River	Quan, Quan	Sci. Total Environ.	2021	37
**37**	Biochar and effective microorganisms promote Sesbania cannabina growth and soil quality in the coastal saline-alkali soil of the Yellow River Delta, China	Cui, Qian	Sci. Total Environ.	2021	31
**38**	Quantitative contributions of climate change and human activities to vegetation changes over multiple time scales on the Loess Plateau	Shi, Shangyu	Sci. Total Environ.	2021	31
**39**	Coupling coordination degree of production, living and ecological spaces and its influencing factors in the Yellow River Basin	Li, Jiangsu	J. Clean Prod.	2021	30
**40**	Spatial-temporal dynamics of agricultural drought in the Loess Plateau under a changing environment: Characteristics and potential influencing factors	Han, Zhiming	Agric. Water Manage.	2021	23
**41**	Land Use Transition and Driving Forces in Chinese Loess Plateau: A Case Study from Pu County, Shanxi Province	Huang, Han	Land	2021	16

Supplementary Table S2. TOP 20 Highly cited scientific papers in the eco-environment research in the Yellow River Basin from CNKI (in English)

NO.	Title	First Author	Journal	Year	Times
**1**	The effect of land use change on the regional environment in the YangJuanGou catchment in the loess plateau of China	Fu, Bojie	Acta Geographica Sinica	1999	1145
**2**	Analysis of impact of human activity on landscape structure in Yellow River Delta——A case study of Dongying Region	Chen, Lixiang	Acta Ecologica Sinica	1996	893
**3**	Quantitative Study on Spatial Variation of Soil Erosion in a Small Watershed in the Loess Hilly Region	Jiang, Shanzhong	Journal of Soil Erosion and Soil Conservation	1996	670
**4**	*Impacts of climate change and human activities on vegetation cover changes in the Loess Plateau*	Xin, Baozhong	Scientia Sinica (Terrae)	2007	549
**5**	Regional Ecological Risk Assessment of Wetland in the Huanghe River Delt	Xu, Xuegong	Acta Scientiarum Naturalium Universitatis Pekinensis	2001	519
**6**	Effects of forest on water circle on the Loess Plateau	Li, Yushan	Journal of Natural Resources	2001	470
**7**	Process-based soil erosion and sediment yield model in a small basin in the Hiliy Loess Region	Cai, Guoqiang	Acta Geographica Sinica	1996	393
**8**	Effects of land use and vegetation restoration on soil quality in a small catchment of the Loess Plateau	Gong, Jie	Chinese Journal of Applied Ecology	2004	383
**9**	Soil water resources and afforestation in Loess Plateau	Yang, Wenzhi	Journal of Natural Resources	2001	377
**10**	Changes of pan evaporation in the recent 40 years over the Yellow River Basin	Qiu, Mingfa	Journal of Natural Resources	2003	376
**11**	Spatiotemporal variability of Soil Moisture in Small Catchment on Loess Plateau--Semivariograms	Wang, Jun	Acta Geographica Sinica	2000	376
**12**	Effect of Vegetation Restoration on Soil Nutrient Changes in Zhifanggou Watershed of Loess Hilly Region	Wang, Guoliang	Bulletin of Soil and Water Conservation	2002	372
**13**	Spatial Heterogeneity of Soil Nutrients in a Small Catchment of the Loess Plateau	Wang, Jun	Acta Ecologica Sinica	2002	369
**14**	Long-term trend of precipitation in the Yellow River basin during the past 50 years	Xu, Zongxue	Geographical Research	2006	364
**15**	Effect simulations of land use change on the runoff and erosion for a Gully Catchment of the Loess Plateau, China	Fu, Bojie	Acta Geographica Sinica	2002	354
**16**	Comparison of grain-size distribution of Red Clay and Loess-paleosol deposits in Chinese Loess Plaeat	Lu, Huayu	Acta Sedimentologica Sinica	1999	321
**17**	Landscape pattern of rural residential areas in Yellow River Delta in recent 20 years	Cai, Weimin	Resources Science	2004	313
**18**	*Influence of land use structure on soil nutrient distribution in loess hilly region*	Fu, Bojie	Chinese Science Bulletin	1998	313
**19**	Soil ecological stoichiometry under different vegetation area on loess hilly-gully region	Zhu, Qiulian	Acta Ecologica Sinica	2013	305
**20**	The study of salt-affected soils in the Yellow River Delta based on Remote Sensing	Guan, Yunxiu	National Remote Sensing Bulletin	2001	298
